# Pilot Study of Sacroiliac Joint Dysfunction Treated with a Single Session of Fascial Manipulation^®^ Method: Clinical Implications for Effective Pain Reduction

**DOI:** 10.3390/medicina57070691

**Published:** 2021-07-06

**Authors:** Dennis Bertoldo, Carmelo Pirri, Barbara Roviaro, Luigi Stecco, Julie Ann Day, Caterina Fede, Diego Guidolin, Carla Stecco

**Affiliations:** 1 Fascial Manipulation Institute by Stecco, 35129 Padua, Italy; info.dennisbertoldo@gmail.com (D.B.); luigi.stecco@teletu.it (L.S.); 2 Department of Neurosciences, Institute of Human Anatomy, University of Padova, 35121 Padua, Italy; caterina.fede@unipd.it (C.F.); diego.guidolin@unipd.it (D.G.); carla.stecco@unipd.it (C.S.); 3 School of Physiotherapy, University of Verona, 37129 Verona, Italy; barbara.roviaro@gmail.com; 4 Azienda Ulss 6 Euganea, Territorial Rehabilitation Unit, 35100 Padova, Italy; lavitava@gmail.com

**Keywords:** fascia, sacroiliac joint dysfunction, pain, Fascial Manipulation, manual treatment, connective tissue, low back pain, thoracolumbar fascia

## Abstract

*Background and Objectives:* Sacroiliac joint dysfunction (SIJD) generally refers to pain in the lower back due to abnormal sacroiliac joint movement, either from hypomobility or hypermobility. It is considered to be the principal cause in up to 40% of low back pain cases. In literature, it emerges that the “fascia”, by its anatomical continuity, if altered or densified in different regions of the body with respect to the sacroiliac joint and its surroundings, may have a fundamental role in the genesis of SIJD and low back pain. The purpose of the present study is to evaluate the effectiveness of incorporating a single session of Fascial Manipulation^®-^Stecco method^®^, treating the muscular fasciae at distance from the painful region. *Materials and Methods*: Twenty patients with acute and chronic sacroiliac joint dysfunction (SIJD) were recruited (16 males and 4 females, mean age of 46.6 ± 12.98 years). Patients underwent a predefined assessment protocol, followed by an evaluation of myofascial pain and subsequent manipulation of the fascia at points at least 20 cm away from the posterior inferior iliac spines (PIIS). Each patient underwent three pain evaluations: pre-treatment (t0), post-treatment (t1), and at a 1-month follow-up (t2). For the evaluation in t0, t1 the numerical rating scale (NRS) for the intensity of pain and the algometer for the pain threshold at the PIIS were used; in t2 only the NRS scale. *Results*: The results obtained by comparing the algometer measurements with the NRS values between t0 and t1 were in both cases statistically significant (*p* < 0.0001), whereas the comparison between the NRS values at t1 and at t2 was not statistically significant (*p* > 0.05). *Conclusions*: A single Fascial Manipulation treatment, even when applied at least 20 cm from the PIIS, can potentially decrease pain around the SIJ. The inclusion of this type of approach in SIJD can allow for improved patient management, better tolerance for other treatments and a more rapid application of pain-free exercise programs.

## 1. Introduction

Sacroiliac joint dysfunction (SIJD) generally refers to pain in the lower back due to abnormal sacroiliac joint movement, either from hypomobility or hypermobility. It is considered to be the principal cause in up to 40% of low back pain cases [1]. The sacroiliac joint (SIJ) is an amphiarthrosis, 75% of which is formed by a synovial joint and the remaining part by fixed fibrocartilaginous elements. The primary stabilizers of the SIJ include the sacroiliac, sacrotuberous and sacrospinous ligaments. Gluteus maximus and medius, the erector spinae, latissimus dorsi, biceps femoris, iliacus, psoas and piriformis, together with abdominal muscles, such as the obliques and transversus abdominis, all provide muscular support for this joint and they are sustained by the thoracolumbar fascia [2]. The thoracolumbar fascia connects the latissimus dorsi muscle with the gluteus maximus muscle and then continues with the deep fascia of the limbs [3,4,5]. Vleeming [6] clearly demonstrates how this aponeurotic structure anchors itself to other bony, ligamentous and muscular structures in the sacroiliac region and also provides a continuum with the fasciae of the lower limbs. Due to this anatomical fascial continuity, altered or densified fascia located in different regions of the body with respect to the sacroiliac joint and its surroundings may have a fundamental role in the genesis of SIJD and low back pain. Furthermore, an average 20% decrease in the deformation of the thoracolumbar fascia during passive lumbar flexion has been detected in patients affected by chronic non-specific low back pain compared to healthy subjects, indicating a correlation between low back pain and an intrinsic alteration within fascia [7].

Concerning low back pain, it should be noted that in 2018 the medical journal ‘The Lancet’ published three articles that voiced alarm with regards to the fact that, on a global level, the years lived with disability caused by low back pain increased by 54% between 1990 and 2015, and that low back pain is now the leading cause of disability worldwide [8].

According to Al-subahi et al. [9], tenderness, or pain felt on palpation of the SIJ, is a reliable sign that the SIJ is the source of pain. The particular algometer that was chosen for this study has already been validated for different types of musculoskeletal pain, such as neck pain and sacroiliac pain [10,11].

The choice of treatment was one session of Fascial Manipulation^®^-Stecco method^®^. This method was chosen because it is based on the anatomical continuity of the fascia and the interaction of tensional lines of force through the fascial system. The application of these principles allows for treatment to be performed at a distance from the painful region, thereby avoiding irritation of locally inflamed tissue while restoring an overall tensional balance. The method entails an initial complete anamnesis, followed by specific movement and palpatory tests and it emphasizes a clinically reasoned biomechanical assessment of the fascial system to understand its role in movement system dysfunctions [12,13]. The physiotherapist then seeks out altered or densified points by manually palpating small codified areas of the deep muscular fascia, known as Centers of Coordination and Centers of Fusion, located within the subjects’ muscular fascia. These small areas are commonly at a distance from reported areas of pain because they are located where tensional vectors are presumed to intersect. Based on Fascial Manipulation^®^ protocol and the integrated biomechanical model applied in this method [12], the therapist then uses their elbow or knuckle to manipulate a select combination of these altered points until solution of the densification is achieved. It has been suggested that the friction produced by the manipulation generates localized heat and that, due to the thermosensitive characteristics of the fasciae, this heat facilitates the passage of hyaluronan within the extracellular matrix (ground substance) from a densified state to a more fluid state, restoring the gliding proprieties of the fasciae [13,14]. Several recent studies have reported that appropriate release of these codified areas appears to result in pain reduction and improved functional and perceived well-being outcomes in low back pain patients [15,16,17].

From the rehabilitation viewpoint, the dysfunctions that can lead to SIJD include muscular, nervous, osseocartilaginous, tendinous and, lastly, visceral origins. With respect to the fascial system, it emerges in literature that the “fascia” is the only structure that connects all these tissues together. However, no previous study has analysed the effectiveness of incorporating a single session of a specific myofascial treatment among the available therapeutic options for SIJD. Accordingly, the aim of this was to assess whether sacroiliac pain could be reduced by Fascial Manipulation^®^, treating the muscular fasciae at distance from the painful region. Perceived pain was measured before and after a single session of myofascial treatment by means of the Numeric Rating Scale and the use of an algometer at the level of posterior inferior iliac spine (PIIS).

## 2. Materials and Methods

The design of this study was a controlled clinical trial lasting six-months. It was a pragmatic study [18,19,20], specifically designed to evaluate the effectiveness of interventions in real-life routine practice conditions. Pragmatic studies are designed to test interventions in the full spectrum of everyday clinical settings in order to maximize applicability and generalizability [18,19,20]. Informed consent was obtained from participants in this study. The inclusion criteria included a sacroiliac pain syndrome related diagnosis or sacroiliitis or pelvic osteoarthritis referred by a medical physician with a target population between 18 and 70 years of age, all acuity levels (i.e., acute, sub-acute, and chronic), with any low back pain related diagnoses. The testing location for this study was an outpatient clinic in the North of Italy. Subjects were recruited for this study on the patient’s first day of physical therapy services. Subjects were excluded from the study if they had a fever, neurological signs of spinal stenosis, loss of reflexes or dysesthesia, structural lesions such as spondylolisthesis or vertebral and sacral fractures, vertebral lesions of a neuroplastic origin, abdominal aneurysms, systemic rheumatological pathologies such as ankylosing spondylitis, lesions of the central or peripheral nervous systems, pregnancy, cardiorespiratory pathologies and psychiatric disorders; or if they were ongoing pharmacological treatment including non-steroid, anti-inflammatory, corticosteroid, painkiller, antidepressant, anti-anxiety or neuroleptic medications. Totally, twenty subjects (4 females, 16 males; age range 22–67 years) were recruited.

### 2.1. Evaluation and Outcome Indicators

**Figure 1:**
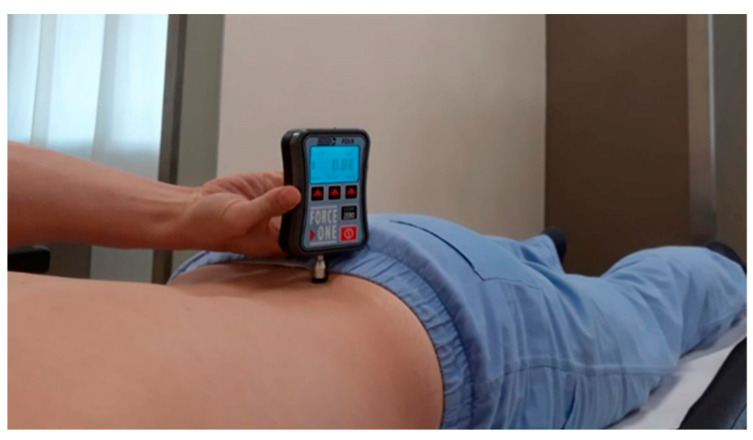
Algometer positioned at the level of the posterior inferior iliac spine (PIIS).

With regards to pain, two outcome measures were applied:

(1) Pain intensity on the Numerical Rating Scale (NRS) was recorded both immediately before and after treatment and at a one-month follow up that was conducted via telephone.

(2) An algometer (WAGER FDIX multi-capacity digital force gauge) produced by Wager Instruments (Accuracy ± 0.2% Dedicated, ± 0.3% interchangeable; Wager Instruments, Greenwich, CT, USA.) was used to measure pain perceived at the level of the right and left posterior inferior iliac spines (PIIS) before and after treatment (Figure 1). With the subjects in the prone position, two algometer measurements were taken before and after treatment at the level of the PIIS. All measurements were recorded at intervals of 30 s. The subjects were instructed to report when the sensation of ‘merely pressure’ changed to the ‘beginning of pain’.

All initial data and subsequent evaluations were collected by the same physiotherapist (DB), while all treatments were performed by a second physiotherapist (LS), who has extensive experience in Fascial Manipulation^®^.

### 2.2. Treatment

The choice of treatment was one session of Fascial Manipulation^®-^Stecco method^®^ [13]. According to Fascial Manipulation^®^ procedure, following a complete anamnesis, including chronology of any previous traumatic events, fractures, significant musculoskeletal dysfunctions, scars and surgical operations, specific movement and palpatory tests are performed. Based on the information gathered and in reference to the Stecco model for interpreting fascial system dysfunction, the physiotherapist then selects an individual combination of densified Centers of Coordination (CC), which are located over muscles, and/or Centers of Fusion (CF), which are located at the periarticular level, to be treated in the single session of Fascial Manipulation^®^. In this particular study, the only limitation was to always treat CC and/or CF that were located at a distance of at least 20 cm from the principal pain area, the PIIS, throughout the treatment. The CC and/or CF points that were treated in all of the single treatment sessions were recorded.

### 2.3. Data Analysis

Statistical analysis of the data was carried out using the GraphPad PRISM 8.4.2 (GraphPad Software Inc., San Diego, CA, USA). The sample size was calculated by G power 3.1 according to Cohen’s d and interpretated as small (d = 0.20), medium (d= 0.50) and large (d = 0.80) [21]. For both algometer and NRS, the respective effect sizes were d = 1.12 (algometer) and d = 0.99 (NRS), resulting in a first our unpublished study, α err prob = 0.05, power: 1-β err prob = 0.95, leading respectively to a sample size of 11 for algometer and 13 for NRS. The normality assessment was carried out using Shapiro-Wilk test. Descriptive statistics were calculated, including measures of central tendency and their dispersion ranges using the mean and the standard deviation (SD) to describe parametric data. A Paired t test was used to analyze data taken with the algometer before (t0) and after (t1) treatment. Moreover, Kruskall-Wallis test was used to analize the NRS scores at t0, t1, and at 1-month follow up, followed by Dunnett’s multiple comparisons test for multiple comparisons. Pearson’s correlation coefficient was used to evaluate a possible correlation between algometer measurements and the NRS scores. The reported results were obtained using a 95% confidence interval, which corresponds to a level of significance of *p* < 0.05.

## 3. Results

### 3.1. Descriptive Data of the Patients

Weight, height, Body Mass Index (BMI), profession and sporting activities, chronicity of sacroiliac pain were recorded for all subjects (Table 1). Chronic SIJ pain was considered to be pain that was present for at least 3 months.

All subjects were given an information sheet and they gave their consent to sharing their health data according to Italian law (article.13, d.lgd. n. 196/2003) with regards to the protection of personal data. They also signed an informed consent to physiotherapy treatment (Rev.02, 22 October 2013).

The mean values of algometer measurements taken at the right and left PIIS of each subject before (t0) and after treatment (t1) are reported in Table 2.

According Paired *t* test, it emerged that:

- mean algometer values at the right PIIS between t0 and t1 (1st and 3rd columns, Table 2.) were statistically significant (*p* < 0.0001), with 95% confidence interval varying from −1.873 to −0.7537 and a correlation coefficient of 0.8604.
- mean algometer values at the left PIIS between t0 and t1 (2nd and 4th columns, Table 2.) were statistically significant (*p* < 0.0001), with 95% confidence interval varying from −1.521 to −0.6430 and a correlation coefficient of 0.8813.
- comparison between mean algometer values taken at the right and left PIIS at t0 (40 measurements; 1st and 2nd columns, Table 2) and mean algometer values taken at the right and left PIIS at t1 (40 measurements; 3rd and 4th columns, Table 2) was statistically significant (*p* < 0.0001), with 95% confidence interval varying from −1.539 to −0.8564 and a correlation coefficient of 0.8636.

**Figure 2:**
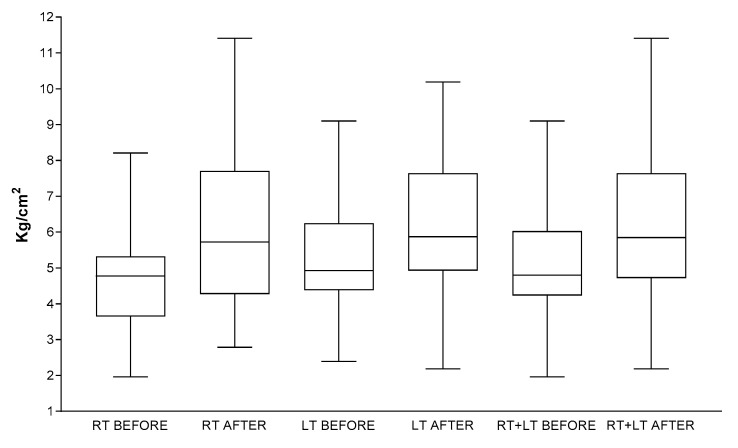
Distribution of the mean values of algometer measurements. The last two box-plots on the right represent the mean values of measurements taken at right and left PIIS before (t0) and after (t1) treatment. Abbreviations; RT = right; LT = left; RT + LT = right + left.

The distribution of the mean values of algometer measurements before and after treatment are reported in Figure 2.

### 3.2. Evaluation of Pain with Numerical Rating Scale (NRS) Scale

The NRS scores measured before treatment (t0), after treatment (t1) and at the 1-month follow up (t2) are reported in Table 3.

By utilizing the Kruskal-Wallis test to compare NRS pain scores, followed by Dunnett’s multiple comparisons test for multiple comparisons it emerged that:

- the comparison between t0 and t1 was statistically significant, (*p* < 0.0001);
- the comparison between t0 and t2 was statistically significant, (*p* < 0.0001);
- the comparison between t1 and t2 was not statistically significant, (*p* > 0.05).

### 3.3. Correlation between Algometer Measurements and NRS

**Figure 3:**
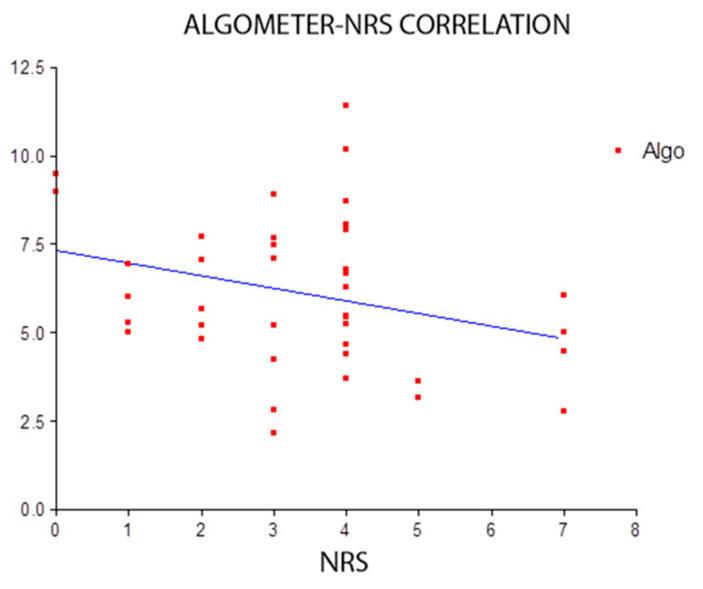
Correlation between Numerical Rating Scale (NRS) scores and algometer measurements, taken at the Posterior Inferior Iliac Spine (PIIS), was not statistically significant (*p* > 0.05).

To conclude the statistical analysis regarding pain, the Pearson’s test was used to evaluate a possible correlation between the values obtained from algometer measurements and those of the NRS or, in other words, whether the NRS scores mirrored the algometer measurements or vice versa. The result was not statistically significant with a score of −0.2981, 95% confidence interval included values between −0.5578 and 0.01491 (Figure 3).

### 3.4. Analysis of Treated Points

**Figure 4:**
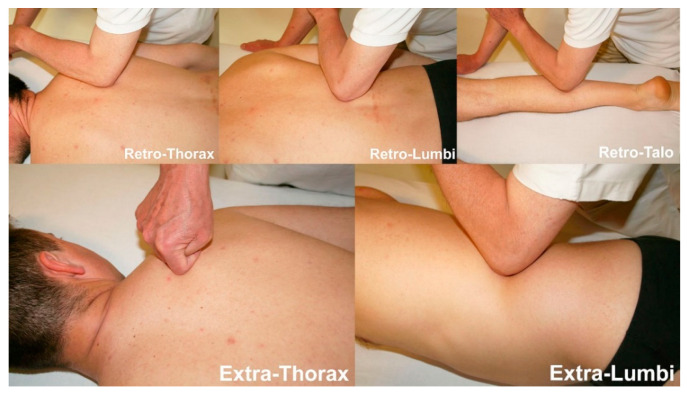
Examples of the location of some of the Centers of Coordination (CC), namely, retro-lumbi, retro-talus, retro-thorax, extra-thorax and extra-lumbi and the treatment modalities applied in these points.

From the analysis of the Centers of Coordination and Fusion (CC/CF) that were manipulated in each session, it emerged that for all subjects the more common points to require treatment were the following CC (Figure 4):

- 11 subjects required treatment of the retro-lumbi (re-lu) CC, which are located over the muscle bellies of the erector spinae at the T12- L 1 level;
- 10 subjects required treatment of the retro-talus (re-ta) CC, which are located in the myotendinous passage of the gastrocnemius, at approximately halfway on the lower leg;
- 7 subjects required treatment of the retro-thorax (re-th) CC, which are located over the muscle bellies of the erector spinae, at the level of T4-T5;
- 7 subjects required treatment of the extra-thorax (ex-th) CC, which are located medially to the scapula, at the level of the scapular spine, over the rhomboid and serratus posterior superior muscles;
- 7 subjects required treatment of the extra-lumbi (ex-lu) CC, which are located below the 12th rib, over the insertion of the oblique muscles.

**Figure 5:**
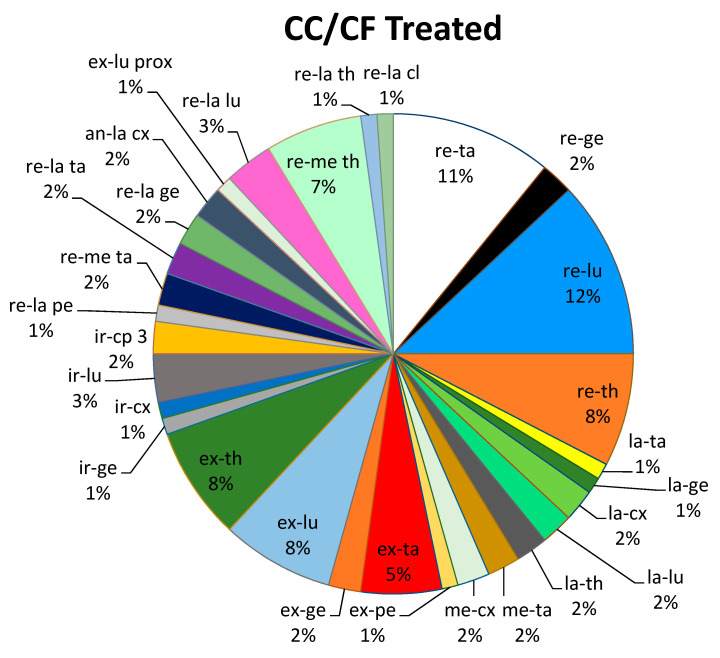
The treated Centers of Coordination (CC) or Centers of Fusion (CF) were all located at a distance of at least 20 cm from the area (PIIS) where algometer measurements were taken. 26% of the points were located in the lumbar region (lu), 26% in the thoracic region (th), 25% in the lower leg (ta), 7% in the thigh (cx), 10% at the level of the knee (ge) and 3% in the foot (pe) and only 3% were in the cervical and head regions (cl, cp).

The following graph (Figure 5) represents the percentages of all points that were treated in all subjects during the single treatment sessions.

## 4. Discussion

All subjects reported a decrease in pain at t1 (after treatment) following a single session of Fascial Manipulation^®^-Stecco method^®^. Only one subject reported a reduction of 1 point on the NRS at t1, while the remaining 19 subjects had a reduction of ≥2 points at t1, with a mean reduction of 3.4 points (t0 ± 6.75; t1 ± 3.35) and an overall (*p* < 0.0001). The comparison between NRS scores at t1 and at t2 (1-month follow up) was not statistically significant (*p* > 0.05) indicating that the reduction in pain had been maintained at the 1-month follow up. The comparison of mean algometer values taken at the right and left PIIS at t0 and t1 was statistically significant (*p* < 0.0001). The Pearson’s test used to evaluate a possible correlation between the values obtained from algometer measurements at the PIIS and those of the NRS was not statistically significant (*p* > 0.05). This result is most likely due to the limited numbers of patients (20) in this study.

The reduction in overall pain (evaluated by means of NRS) and local pain (measured by an algometer at the PIIS) was obtained without treating the painful area directly. Based on anatomical fascial continuity, all treatments were performed on fascial tissues that were at a distance of at least 20 centimetres from the painful area. This result suggests that, in clinical practice, it is possible to improve symptoms by working at a distance from the painful area. This is advantageous because it permits a safer treatment that avoids irritating potentially inflamed areas and, at the same time, promotes a faster physiological recovery presumably due to the restoral of correct fascial tensioning.

In this study, the chosen method of treatment, namely Fascial Manipulation^®^- Stecco method^®^, evaluates each subject according to their individual clinical history, including any previous trauma. Rather than limit treatment to the painful region, which may lead to a temporary outcome, it focusses on fascial continuity and the connections between fascia and other tissues in order to identify the primary cause of dysfunction.

Vleeming [22] explains how the deep fascia can effectively transmit force over distances and that, in particular, in reference to the low back region, it is the posterior layer of the thoracolumbar fascia that carries out this function. Moreover, the force closure in SIJ occurs because of altered joint reaction force via taut ligaments, muscles, ground reaction force they are reacting to at the moment, but also at the fascial tension, as Vleeming [23] reported. Whenever the fascia is densified, it could modify the lines of force within the fascial tissue causing a change in the basal tension of the thoracolumbar fascia. This could stimulate the mechanoreceptors and nociceptors embedded in this fascia [24], resulting in a biomechanical imbalance at the sacroiliac and lumbosacral joints with subsequent inflammation, movement dysfunction and pain. According to Fascial Manipulation^®^ concepts, joint pain can be seen as a consequence of a biomechanical imbalance due to alterations in fascial density. The implication is that a systematic biomechanical assessment of the fascial system can lead to applications of myofascial treatments, such as Fascial Manipulation^®^, that can restore the correct density of the hyaluronan between the layers of the thoracolumbar fascia and in adjacent fascial layers and, in this way, re-establish correct basal tension, receptor transmission and sacroiliac function, with a consequent reduction in perceived pain [13]. Furthermore, the possibility of rapidly decreasing pain around the SIJ can ostensibly improve patient management, allow for better tolerance of other treatments and permit the early application of pain-free exercise programs to establish more efficient movement patterns [1,9,25].

In this study, pain reduction was greater in subjects with chronic low back pain, indicating that fascial tensioning was a key element in maintaining incorrect joint alignment. While all subjects with acute low back pain cases reported a decrease in pain on the NRS, the immediate post-treatment algometer measurement improvements were not statistically significant. This could imply the presence of an acute inflammation in the sacroiliac joints, which clearly cannot resolve immediately. In such cases, the aim is to improve fascial tensioning in order to facilitate the resolution of inflammation in the period following treatment.

This study had several limitations. The patient cohort used in this study is small, with only 20 subjects with SIJD being assessed. This number did not allow us to evaluate the efficacy of the treatment and to correlate NRS and algometer measurements. The lack of algometer measurements at the follow up, due to organizational difficulties, is another limitation.

## 5. Conclusions

The results obtained in this study appear to support the hypothesis that fascia could be implicated in the aetiology of non-specific low back pain, in particular SIJD. Analysis of dysfunction according to fascial continuity throughout the entire body may help to understand symptoms more effectively, leading to treatments that are aimed more at restoring biomechanical balance within the fascial system, rather than treatments that are merely aimed at the localized symptoms. An algometer appears to be a valid instrument for evaluating treatment results, particularly in chronic cases.

The inclusion of this type of approach in SIJD can allow for improved patient management, better tolerance for other treatments and a more rapid application of pain-free exercise programs.

## Figures and Tables

**Table 1:** Descriptive data of the patients.

Patient	Male\Female	Age (Years)	Weight (kg)	Height (m)	BMI	Type of SIJ Pain	Time	Sport
1	F	39	60	1.6	23.4	chronic	1 years	no
2	M	37	83	1.9	23	chronic	1 years	aikido
3	M	67	64	1.69	22.4	acute	5 days	no
4	M	40	85	1.93	22.8	chronic	1 years	tennis
5	M	42	79	1.77	25.2	chronic	5 years	no
6	M	59	93	1.87	26.6	acute	2 weeks	walk
7	F	35	69	1.6	27	chronic	15 years	yoga
8	F	67	62	1.63	23.3	chronic	6 months	no
9	M	52	97	1.78	30.6	acute	5 days	no
10	F	22	50	1.65	18.4	chronic	5 years	volleyball
11	M	22	65	1.7	22.5	chronic	4 months	football
12	M	46	94	1.7	32.5	chronic	1 years	no
13	M	40	96	1.8	29.6	chronic	1 years	no
14	M	48	94	1.81	28.7	chronic	10 years	no
15	M	55	75	1.77	23.9	chronic	10 years	running
16	M	55	83	1.86	24	chronic	8 months	ski
17	M	61	98	1.78	30.9	chronic	25 years	no
18	M	56	59	1.67	21.2	chronic	30 years	no
19	M	52	75	1.9	20.8	chronic	5 years	no
20	M	36	71	1.89	19.9	acute	2 weeks	gym

**Table 2:** Mean values and standard deviation of algometer measurements taken at the level of the right (rt) and left (lt) posterior inferior iliac spine (PIIS) in 20 subjects. The first and second columns report mean values at t0, while the third and fourth columns report mean values taken at t1. TX = treatment.

Patients	RT BEFORE TX (kg/cm^2^)	LT BEFORE TX (kg/cm^2^)	RT AFTER TX (kg/cm^2^)	LT AFTER TX (kg/cm^2^)
1	3.46 ± 0.06	4.80 ± 0.03	3.73 ± 0.10	5.26 ± 0.10
2	6.45 ± 0.13	5.75 ± 0.04	7.10 ± 0.10	7.50 ± 0.10
3	4.27 ± 0.01	2.39 ± 0.10	5.04 ± 0.02	5.29 ± 0.02
4	6.23 ± 0.04	7.23 ± 0.10	7.91 ± 0.20	8.74 ± 0.10
5	4.76 ± 0.10	6.56 ± 0.10	8.93 ± 0.03	7.70 ± 0.10
6	8.16 ± 0.01	9.10 ± 0.10	11.41 ± 0.20	10.19 ± 0.04
7	4.49 ± 0.04	4.37 ± 0.02	8.07 ± 0.10	6.80 ± 0.10
8	4.80 ± 0.10	4.75 ± 0.10	5.21 ± 0.20	4.83 ± 0.20
9	2.43 ± 0.21	2.40 ± 0.30	4.23 ± 0.10	5.22 ± 0.21
10	1.96 ± 0.01	4.72 ± 0.33	2.79 ± 0.10	4.47 ± 0.11
11	5.24 ± 0.12	5.05 ± 0.10	5.43 ± 0.20	5.47 ± 0.01
12	8.21 ± 0.10	8.26 ± 0.20	8.98 ± 0.03	9.49 ± 0.02
13	4.22 ± 0.02	4.55 ± 0.20	4.40 ± 0.21	4.69 ± 0.10
14	5.26 ± 0.14	6.12 ± 0.20	5.02 ± 0.10	6.06 ± 0.10
15	4.29 ± 0.20	5.67 ± 0.40	6.02 ± 0.10	6.95 ± 0.40
16	5.35 ± 0.23	4.41 ± 0.30	7.06 ± 0.30	5.68 ± 0.10
17	5.17 ± 0.30	6.29 ± 0.20	7.07 ± 0.21	7.72 ± 0.10
18	2.10 ± 0.17	2.41 ± 0.30	2.84 ± 0.13	2.19 ± 0.10
19	2.69 ± 0.20	2.83 ± 0.10	3.19 ± 0.20	3.62 ± 0.30
20	4.90 ± 0.20	5.23 ± 0.10	6.29 ± 0.01	6.69 ± 0.04
**Mean ± SD**	**4.72 ± 1.73**	**5.14 ± 1.84**	**6.03 ± 2.30**	**6.22 ± 1.97**

**Table 3:** Numerical Rating Scale (NRS) scores. The before treatment (t0) NRS scores are reported in the first column, the after treatment (t1) NRS scores are in the second column and the NRS scores at the 1-month follow up (t2) are reported in the third column. TX = treatment.

Patients	NRS before TX (t0)	NRS after TX (t1)	NRS 1 Month (t2)
1	6	4	1
2	7	3	2
3	7	1	0
4	6	4	1
5	8	3	1
6	8	4	1
7	8	4	7
8	6	2	0
9	7	3	2
10	8	7	7
11	6	4	6
12	5	0	1
13	5	4	5
14	10	7	8
15	5	1	3
16	5	2	5
17	7	2	2
18	7	3	3
19	7	5	4
20	7	4	5
Mean ± SD	***6.80 ± 1.30***	**3*.40 ± 1.80***	**3.20 *± 2.51***

## Data Availability

The data presented in this study are available in the article.
